# A systematic review of the implications of lipocalin-2 expression in periodontal disease

**DOI:** 10.1038/s41432-024-01070-y

**Published:** 2024-11-08

**Authors:** Diana L. Solís-Suárez, Saúl E. Cifuentes-Mendiola, Ana L. García-Hernández

**Affiliations:** 1 Laboratory of Dental Research, Section of Osteoimmunology and Oral Immunology, FES Iztacala, National Autonomous University of Mexico, 54714 Mexico, Mexico State Mexico; 2 Postgraduate Course in Dental Sciences. National Autonomous University of Mexico, Mexico City, Mexico

**Keywords:** Periodontitis, Oral medicine

## Abstract

**Objective:**

Evidence suggests that lipocalin-2 (LCN-2), a bone-derived protein, is upregulated in periodontal diseases. This systematic review aimed to evaluate LCN-2 concentrations in individuals with periodontal diseases, identifying the most suitable body fluids for its detection, the type of periodontal disease with the highest LCN-2 expression, its association with other inflammatory markers and systemic diseases, and whether its expression can be modified by periodontal treatment.

**Methods:**

A systematic search of Google Scholar, PubMed, and ProQuest up to August 2024 was conducted. The studies were screened and selected by the authors according to specific eligibility criteria. Quality assessment of the included studies was performed according to the study type using STROBE statement for observational studies or the modified Jadad scale for experimental studies. The review was registered in PROSPERO (CRD42023458565).

**Results:**

In total, three thousand six hundred and thirty-eight reports were identified, of which twenty-seven were full-text assessed for eligibility, including eleven articles. Seven articles were observational, and four were experimental. Significantly elevated LCN-2 levels were reported in patients with periodontal disease across 9 studies, being higher in periodontitis rather than gingivitis. LCN-2 was mainly detected in gingival crevicular fluid (GCF) and saliva. LCN-2 expression is related to the increment of inflammatory markers, and periodontal therapy decreases LCN-2 concentrations. LCN-2 levels were aggravated when periodontitis was accompanied by obesity and type 2 diabetes.

**Conclusion:**

LCN-2 is implicated in periodontal diseases, probably through the inflammation process.

Key points

- Increased LCN-2 expression is related to periodontal proinflammatory markers detected in GCF and saliva.
- Periodontal treatment decreases proinflammatory markers and LCN-2 expression.
- LCN-2 concentrations are even higher in periodontitis accompanied by obesity and type 2 diabetes.

## Introduction

Lipocalin-2 (LCN-2), also known as neutrophil gelatinase-associated lipocalin (NGAL), is mainly produced by neutrophils, osteoblasts, and adipocytes^1^. It was first described as a 25-kDa glycoprotein isolated from neutrophil granules that captures bacterial siderophores to promote host resistance to infection^2^. Over the past few years, new knowledge regarding LCN-2 functions in other physiological processes has emerged, including in the regulation of glucose and energy metabolism through appetite suppression^1^ and bone remodeling in response to mechanical forces^3^. Additionally, LCN-2 is considered an inflammatory mediator as it is induced by proinflammatory cytokines IL-6, IFN-γ and TNF-α in adipocytes, neutrophils, and macrophages and is involved in inflammatory pathologies such as obesity, inflammatory bowel disease, type 2 diabetes, and others^4^.

LCN-2 has also been associated with periodontal diseases^5^, one of the most prevalent pathologies in oral cavity diseases, affecting 19% of the global population^6^. Periodontitis is a clinical condition characterized by destruction of the gingiva and alveolar bone and tooth loss. This condition arises from the host’s immune-inflammatory response triggered by oral dysbiosis in susceptible individuals^7^.

In periodontitis, unresolved inflammation leads to tissue injury. The mechanisms driving soft and hard tissue destruction involve the interaction of mediators from both innate and acquired immunity, cytokine networks including IL-1α, IL-1β, TNF-α, IL-6, and IL-17, lipid mediators, and chemokines^8^. Periodontal pocket formation and alveolar bone loss are hallmarks of periodontitis^7^. These processes escalate when excessive numbers of neutrophils are recruited to the gingival crevice but fail to fight the infection. Instead, neutrophils release degradative enzymes, cytotoxic substances, and proinflammatory cytokines for the recruitment of macrophages, dendritic cells, and lymphocytes. This further exacerbates inflammation by the production of additional proinflammatory mediators, and stimulation of T helper cells Th1 and Th17 response. This microenvironment induces higher expression of Receptor Activator on Nuclear Factor kB Ligand (RANKL), promoting the differentiation and activation of bone-resorbing cells (osteoclasts)^9^.

Traditionally, clinical and radiographic parameters were used to diagnose and treat periodontitis, but these methods made it challenging to classify and predict the evolution of the disease. The current classification system of periodontitis is based on the etiology factors, clinical history, and clinical parameters while also emphasizing the use of biomarkers to predict disease risk and progression^10–13^.

Multiple molecules related to inflammation (TNF-α, IL-6), bone resorption (RANKL, osteoprotegerin (OPG), and tissue degradation matrix metalloproteinase (MMP) -8 and MMP-9 have been considered as possible biomarkers of periodontal disease^14^. The expression of these biomarkers has been analyzed in oral fluids such as gingival crevicular fluid (GCF), which provides localized information, and in saliva, which contains both local and systemic markers; and even in extra-oral fluids like serum^15,16^, urine^17–19^, and tears^20^, to study the relationship between periodontitis and systemic diseases.

An increasing number of studies have reported elevated LCN-2 levels in patients with periodontal diseases^5,17,21,22^, suggesting that LCN-2 may play an important role in their pathogenesis, probably through its role in neutrophil-driven inflammation and bone remodeling via osteoblast activity^3,23^.

Furthermore, due to LCN-2 high expression in serum or urine in inflammatory systemic diseases, such as kidney disease, rheumatic diseases, obesity, diabetes, and heart failure, has been proposed as a potential biomarker linking periodontitis and systemic diseases^17,20,24,25^. However, the role of LCN-2 in relation to periodontal inflammatory markers remains unclear. It has not yet been established which body fluids are used for LCN-2 detection, whether its increased expression depends on the type of periodontal disease, or if periodontal treatment reduces LCN-2 expression. It is unknown if LCN-2 expression is associated with both periodontitis and systemic diseases. Therefore, this systematic review aimed to examine the clinical evidence regarding LCN-2 expression to determine its implications in periodontal disease.

## Methods

The study was performed in accordance with Cochrane standards for systematic reviews. The search criteria complied with the PRISMA 2020 guidelines^21^. The protocol was registered at the International Prospective Register of Systematic Reviews (PROSPERO) under the number CRD42023458565. To determine the implications of LCN-2 expression in periodontal disease, we addressed the following questions:

1. Is there an increase in LCN-2 concentrations in periodontal disease?
2. Which type of periodontal disease shows the highest LCN-2 expression?
3. In which body fluid has LCN-2 been detected?
4. Are LCN-2 levels associated with other inflammatory biomarkers?
5. Does periodontal treatment modify LCN-2 concentrations?
6. Are LCN-2 concentrations higher when a systemic disease accompanies periodontal disease?

### Eligibility criteria

The criteria for considering studies for this systematic review were defined based on the elements of the Patient population, Intervention, Comparative controls, Outcome(s), and Studies, also known as the PICOS model^26^, as outlined in Table 1. We included observational (cohort, cross-sectional, case-control) and experimental studies (randomized controlled trials) published from 2000 to 2024. Exclusion criteria included animal or in vitro studies, reviews, case reports, editor letters, meta-analyses, or congress abstracts, studies with insufficient data on LCN-2 concentrations, and studies reporting LCN-2 levels in systemic diseases without periodontitis.

**Table 1:** PICOS criteria for study selection.

Population (P):	Subjects ≥18 years old with gingivitis and/or periodontitis. Due to the heterogeneity of the criteria and the definitions of periodontitis any current and past classifications defined and diagnosed by authors were taken into consideration.
Intervention (I):	Screening tests and studies based on any kind of periodontal therapy.
Control (C):	Periodontally and systemically healthy individuals.
Outcome (O):	Changes in LCN-2 concentrations and at least 3 periodontal health parameters: bleeding on probing, BOP (%); probing depth, PD (mm); clinical attachment loss, CAL (mm); plaque index, PI; and gingival index.
Studies (S):	Observational studies: cohort, case-control, cross-sectional, prospective. Experimental studies: Randomized controlled trials Published from 2000 to 2024.

### Information sources and search strategy

Three electronic databases (Google Scholar, PubMed, and ProQuest) were searched up to August 29^th^, 2024, using the following MeSH/entry terms, synonyms, and free terms in combination with the Boolean operators (OR, AND) to broaden the search results: (lipocalin-2) OR (lipocalin 2) OR (LCN-2) OR (NGAL), AND periodontitis.

The authors (DLSS, SECM, and ALGH) conducted the searches independently, and the selected studies were reviewed to reach a consensus. Duplicated studies were eliminated using EndNote 20. All articles identified through the search were assessed for eligibility criteria. Full texts that were unavailable were excluded after an initial screening of titles and abstracts. Potentially relevant studies were further examined to answer the research question.

### Data collection process

Data were independently extracted by two of the authors (ALGH and DLSS). The selected articles were organized according to their type. The following data were extracted: (1) first author, (2) year of publication, (3) country, (4) purpose of the study, (5) sample evaluation method (6) number of participants, (7) demographic data (sex and age), (8) periodontal condition, (9) mean parameters considered to evaluate periodontal status: Bleeding On Probing (BOP) (%), Probing Depth (PD) (mm), Clinical Attachment loss (CAL) (mm), Plaque index (PI) and Gingival Index (GI, (10) presence of systemic disease (11) biological fluid or tissue used for LCN-2 detection (GCF, saliva, serum, or tears), (12) mean baseline and post-treatment LCN-2 concentrations reported, (13) related inflammatory biomarkers and (14) main outcome. After data extraction, all authors reviewed and discussed the data to reach a consensus.

In cases of missing data, the authors were contacted by email to clarify any information.

### Risk of bias and quality assessment

The quality of the included studies was assessed according to their type. For observational studies, the STROBE Statement was used. This checklist evaluates 22 points that should be included in articles reporting this type of research. Each item evaluated was rated as yes (green), no (red), or unclear (yellow), and a total score was obtained based on the number of “yes” ratings for each study. Quality was considered low if the study scored 0–7 out of 18 points, moderate quality if it scored 8–15 points, and high quality if it scored 16–22 points^27^.

For experimental studies, quality was assessed using the modified Jadad scale for randomized controlled trials, which evaluates randomization, double-blinding, withdrawals and dropouts, inclusion and exclusion criteria, adverse effects, and statistical analysis. Each item was rated as yes (1 point), no (0 points), and not described (0 points). Scores of 4–8 indicate good to excellent quality, while 0–3 indicate poor or low quality^28,29^.

## Results

### Study selection

**Fig. 1:**
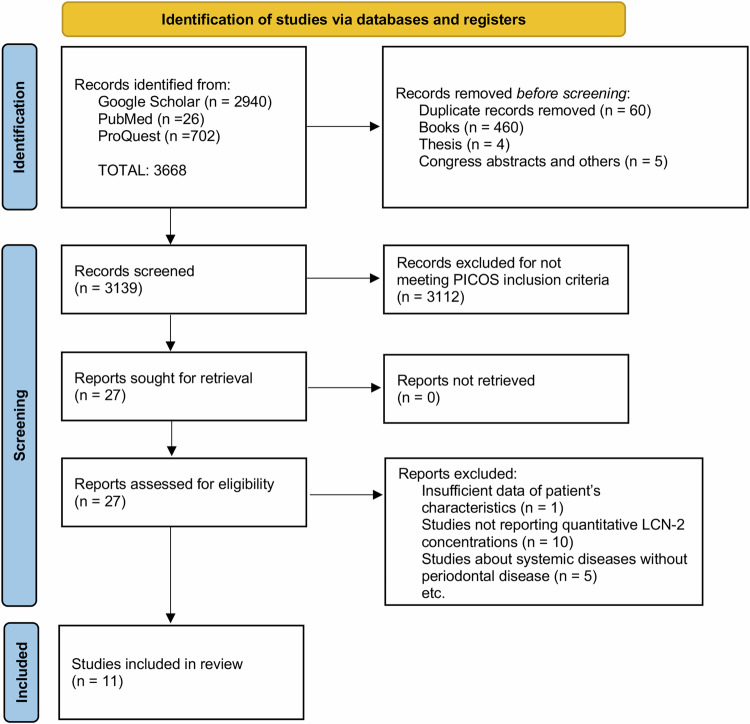
PRISMA flow diagram depicting the study selection process. Study flowchart shows the steps for study slection: identification, screening and finally included studies.

A total of 3668 articles were retrieved using the search strategy described above. After removing duplicate articles (*n* = 60), books (*n* = 460), theses (*n* = 4), congress abstracts, and editor letters (*n* = 5), 3139 articles were screened based on their titles and abstracts. Studies involving animals or in vitro experiments, reviews, and case reports were excluded (*n* = 3112). Full-text articles that did not meet the inclusion criteria were also excluded due to insufficient patient data (*n* = 1), studies that did not use quantitative methods (*n* = 10), and studies on systemic diseases without periodontitis (*n* = 5), resulting in a final total of eleven studies^5,17,20,22,24,25,30–34^. The study flowchart is shown in Fig. 1.

### Study characteristics

#### General study characteristics

Relevant information was collected using data collection sheets and is summarized in Supplementary Table 1. The characteristics of the articles included in the review were first divided by study type. Seven studies were observational, and four were experimental. Six were cross-sectional studies that reported differences in LCN-2 levels between healthy individuals and patients with periodontal diseases^17,20,24,33,34^, one was a prospective cohort study that reported changes in LCN-2 levels at two-time points^32^, and four were prospective randomized controlled trials that evaluated LCN-2 concentrations before and after periodontal treatments^5,22,25,30^.

The studies were published between 2013 and 2023 and originated from seven different countries: Japan (*n* = 2), USA (*n* = 2), India (*n* = 2), Denmark (*n* = 1), Italy (*n* = 1), Turkey (*n* = 2), and Egypt (*n* = 1). The total sample size ranged from 47 to 168, with the number of gingivitis cases ranging from 13 to 34, periodontitis cases from 18 to 101, and control groups from 10 to 52. Nine out of eleven studies included both female and male participants^5,20,22,25,30–34^. The study by Nakajima et al. included only males^17^, while Mahendra et al. did not specify the sex of participants^24^. The age range across studies was from 18 to 80 years.

The definitions and diagnostic criteria for periodontal disease varied considerably across the eleven studies, though all of them specified the participants’ periodontal condition. Only the studies by Tan et al., Aspiras et al., Ceylan et al., and Morelli et al. provided complete data for the five essential parameters for diagnosing periodontal disease (BOP, PD, CAL, PI, and GI)^5,22,32,33^. Five out of eleven studies enrolled individuals with gingivitis^5,22,32–34^, while all eleven studies included participants with periodontitis. Most studies reporting LCN-2 concentrations focus on subjects with periodontitis.

Seven of the included studies reported increased LCN-2 concentrations in patients with periodontal diseases compared to healthy controls^5,17,20,30,32–34^. Of these, only Ceylan et al. and Tan et al. reported an increase in LCN-2 concentrations in subjects with gingivitis^5,33^. At the same time, Morelli et al. found no differences between the control group and gingivitis patients^32^. All seven studies indicated higher LCN-2 levels in periodontitis, particularly in stage III^5,17,20,30,32–34^. These findings suggest that LCN-2 levels increase in proportion to the severity of periodontal disease.

All studies specified the biological fluid used for LCN-2 detection. The fluids were GCF (*n* = 4)^5,20,30,34^, saliva (*n* = 4)^22,31–33^, serum (*n* = 2)^25,33^, urine (*n* = 1)^17^, and tears (*n* = 1)^20^. Only the study by Mahendra et al. assessed LCN-2 levels in subgingival tissue^24^. The study by Belstrøm was the only one to report lower LCN-2 levels in saliva compared to periodontally healthy individuals^31^. GCF and saliva were the most frequently used fluids for LCN-2 detection.

Regarding the sample evaluation method, ELISA was used in seven studies^5,20,24,25,30,33,34^, followed by Multiplex immunoassays in three studies^22,31,32^. Other techniques included Western blot and tandem mass tag labeling^34^.

Three studies assessed LCN-2 levels in subjects with systemic diseases. Pradeep et al. included patients with obesity^20^, Belstrøm et al. included a group with psoriasis^31^, and Mahendra et al. studied subjects with type 2 diabetes^24^. Pradeep et al. and Mahendra et al. reported even higher LCN-2 concentrations when both a systemic disease and periodontitis were present.

Four studies evaluated changes in LCN-2 concentration in response to periodontal therapy. Aspiras et al. investigated the short-term effects of power brushing on gingivitis and periodontitis. Although this study did not show baseline LCN-2 concentrations, they observed that LCN-2 levels remained unchanged between treatments despite improvements in BOP, PD, CAL, PI, and GI induced by power brushing^22^. Isola et al. assessed the efficacy of full-mouth scaling and root planing in stage III periodontitis and observed reductions in BOP, PD, CAL, PI, and LCN-2 concentrations after six months of treatment^25^. Ceylan et al. evaluated LCN-2 concentrations after non-surgical periodontal treatment in stage III periodontitis; improvements in BOP, CAL, PD, PI, and GI were accompanied by lower LCN-2 levels after therapy^5^. Alkayali et al. presented LCN-2 concentrations before and after treatment with scaling and root planing compared to polycaprolactone nanofibers loaded with oxytetracycline hydrochloride and zinc oxide (PCL + OTC + ZNO). Nanofibers were more effective than scaling and root planing in reducing LCN-2 levels and improving PD, CAL, PI, and GI^30^. This evidence suggests that LCN-2 expression can be decreased by periodontal therapy, and the reduction of this protein could be related to the improvement of periodontal inflammation.

Nine out of eleven studies evaluated other inflammatory markers related to periodontal diseases. Three studies reported increased IL-1β levels^22,32,33^, two studies reported elevated MMP-9^32,34^, and one study each reported increased TNF-ɑ and Sema-3 levels^5^ when LCN-2 was also elevated. Additionally, inflammatory markers involved in the pathophysiology of systemic diseases were assessed. For instance, elevated levels of β-macroglobulin, a urinary marker of nephropathy^17^, as well as sensitivity C-Reactive Protein (hs-CRP) and brain natriuretic peptide (NT-proBNP), which are implicated in cardiovascular disease^25^, were observed. In contrast, adiponectin, a hormone that regulates insulin sensitivity, was significantly decreased in diabetic subjects with periodontitis^24^, while LCN-2 levels were increased. These findings demonstrate that LCN-2 is closely related to both periodontal and systemic inflammation.

### Quality assessment

The quality assessment of the included studies is presented in Tables 2 and 3.

**Table 2:**
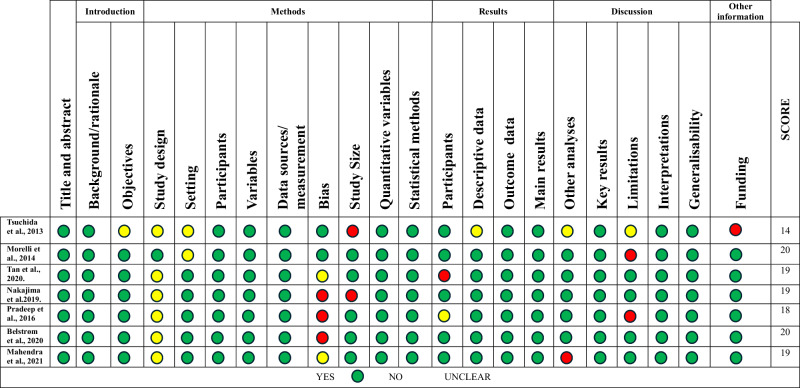
Strobe quality assessment.

**Table 3:** Jadad quality assessment.

	Aspiras et al.^22^ USA	Isola et al.^25^ Italy	Ceylan et al.^25^ Turkey	Alkayali et al.^30^ Egypt
Was the study described as randomized?	Yes	Yes	No	Yes
Was the method of randomization appropriate?	Not described	Yes	Not described	Not described
Was the study described as blinded?	Yes	Yes	No	No
Was the method of blinding appropriate?	Not described	Yes	Not described	Not described
Was there a description of withdrawals and dropouts?	No	Yes	No	No
Was there a clear description of the inclusion/exclusion criteria?	Yes	Yes	Yes	Yes
Was the method used to assess adverse effects described?	No	No	No	No
Was the method of statistical analysis described?	Yes	Yes	Yes	Yes
Scores	4	7	2	3

The STROBE scores for the included observational studies ranged from 14 to 20 points. The study by Tsuchida et al. was the only one considered to be of moderate quality (14 points), as although they clearly defined the study population and groups in the methods section, they presented the data for mild and moderate periodontitis in the same group in the results section^34^. Additionally, they did not justify the sample size, omitted the power description, and inconsistently applied the independent and dependent variables across groups. Furthermore, limitations and funding sources were not described. Despite these shortcomings, we decided to include the study because it contained the raw data necessary to determine LCN-2 levels in periodontal diseases. The rest of the studies were of good quality (Table 2).

The Jadad Scale for randomized controlled trials revealed that the studies by Ceylan et al. and Alkayali et al. were of poor quality (2 and 3 points, respectively) due to inadequate descriptions of randomization, blinding methods, and adverse treatment effects^5,30^. However, we included both studies because they reported LCN-2 concentrations before and after treatment. The studies by Aspiras et al. and Isola et al. were of good quality^22,25^ (Table 3).

## Discussion

To the best of our knowledge, this is the first systematic review that explores the association between LCN-2 concentrations, the stages of periodontal disease, appropriate biological fluids for its detection, its relationship with inflammatory markers and systemic diseases, as well as its responsiveness to treatment comparing individuals with periodontal disease to periodontally healthy individuals.

Due to the heterogeneity of the included studies, a meta-analysis could not be performed. However, this systematic review allowed the inclusion of both observational and experimental studies to assess the implications of LCN-2 expression in periodontal disease.

LCN-2 was found to be overproduced in all cases of gingivitis and almost all cases of periodontitis, suggesting its potential as a marker for periodontal diseases. LCN-2 levels increased proportionally with the severity of the disease despite variations in the source of LCN-2 detection across studies, inconsistencies in diagnostic criteria for periodontitis, and differences in cohort selection by periodontal stage.

LCN-2 concentration varies according to the stage of periodontitis, considering both current and past classifications. Previous research has shown that certain biomarkers increase with the severity of periodontitis such as TNF-α and IL-17^35^. The detection of molecules like hs-CRP and fibrinogen in periodontitis stage III and IV, are useful for predicting the development of cardiovascular disease^35^. However, this pattern does not apply to all markers. For example, Baser et al. found significantly higher GCF IL-1β and prostaglandin E2 (PGE2) levels in severe periodontitis compared to mild cases but no difference in hs-CRP levels in either serum or GCF based on periodontitis severity^36^. This review found that elevated LCN-2 levels were associated with stage I and II periodontitis, while even higher LCN-2 levels in stage III were observed. The results of Belstrøm et al. were inconsistent, highlighting the need for further research^31^. No studies were found that evaluated LCN-2 levels in stage IV periodontitis.

Future studies should explore the effects of inhibiting or regulating LCN-2 concentrations at different stages of periodontitis, along with the measurement of inflammatory markers and periodontal clinical parameters, to understand its role better.

As demonstrated in this review and previous studies, GCF and saliva are the preferred biological fluids for detecting periodontal disease biomarkers^35^. All studies that used GCF reported higher LCN-2 levels in patients with periodontitis compared to controls. However, results from saliva were mixed. Belstrøm et al. found lower LCN-2 levels in periodontitis patients, while Morelli et al. reported significantly higher concentrations in this group compared to healthy individuals^31,32^. A study by Westerlund et al. also identified higher saliva LCN-2 levels in periodontitis patients using western blot, consistent with Morelli’s findings^21,32^. Other biomarkers in saliva have shown similar variability, such as IL-23, which was significantly lower in both localized and generalized periodontitis compared to healthy subjects^37^. No differences were observed in salivary protein carbonyl levels^15^, IL-17^38^, IL-1β, TNF-α, or nitric oxide^39^ between periodontitis patients and healthy controls. Therefore, saliva may not be the most reliable fluid for detecting periodontal disease biomarkers.

Fluids such as serum, urine, and tears were less frequently used for LCN-2 detection. However, these fluids present a valuable option for monitoring changes in inflammatory marker concentrations in response to periodontal treatment and tracking periodontal disease progression based on clinical parameters^35^ For instance, studies conducted by Isola et al., Ceylan et al., Aspiras et al., and Alkayali et al. evaluated LCN-2 levels before and after various periodontal treatments, demonstrating improvements in clinical parameters and reductions in LCN-2 levels^5,22,25,30^. An additional advantage of using extra-oral fluids is that it enables researchers to explore the relationship between periodontitis and systemic diseases^35^. For example, the study by Pradeep et al. assessed LCN-2 levels in both tears and GCF, finding elevated LCN-2 concentrations in both fluids from patients with periodontitis compared to healthy controls. LCN-2 levels were further increased in patients with both periodontitis and obesity, suggesting that this protein may be a key marker in understanding the link between obesity and periodontitis^20^. Similarly, Nakajima et al. evaluated β-macroglobulin alongside LCN-2 concentrations in urine to investigate the connection between periodontitis and nephropathy^17^.

Numerous studies have demonstrated that LCN-2 plays a role in the pathogenesis of significant diseases such as cancer^40^, inflammatory bowel disease^41^, diabetes^42^, cardiovascular disease^43^, and nephropathy^44^, among others. Local inflammation in the periodontium could potentially exacerbate these conditions^45^. In this systematic review, three studies assessed LCN-2 concentrations in individuals with systemic diseases. Pradeep et al. included groups of patients with obesity, Belstrøm et al. studied individuals with psoriasis, and Mahendra et al. focused on patients with generalized periodontitis and type 2 diabetes^20,24,31^. Therefore, further research on the role of LCN-2 in both systemic diseases and periodontitis will be crucial for understanding the connection between these pathologies and uncovering new etiological factors in periodontitis.

## Conclusions

This systematic review suggests that LCN-2 is involved in periodontal diseases probably through inflammation, as LCN-2 levels increase in periodontal diseases and correlate with the type and severity of the condition. GCF and saliva are the most used fluids for its detection. Periodontal treatment helps reduce LCN-2 levels along with other inflammatory markers, contributing to the improvement of periodontal health.

## Supplementary information

SI Table 1

PRISMA Checklist

## Data Availability

The datasets used and analyzed during the current study are available from the corresponding author on reasonable request o in PROSPERO. CRD42023458565

## References

[1] Mosialou I, Shikhel S, Liu J-M, Maurizi A, Luo N, He Z, et al. MC4R-dependent suppression of appetite by bone-derived lipocalin 2. Nature. 2017;543:385–90. 28273060 10.1038/nature21697 PMC5975642

[2] Kjeldsen L, Johnsen AH, Sengeløv H, Borregaard N. Isolation and primary structure of NGAL, a novel protein associated with human neutrophil gelatinase. J Biol Chem. 1993;268:10425–32. 7683678

[3] Rucci N, Capulli M, Piperni SG, Cappariello A, Lau P, Frings-Meuthen P, et al. Lipocalin 2: a new mechanoresponding gene regulating bone homeostasis. J Bone Min Res. 2015;30:357–68. 10.1002/jbmr.2341 25112732

[4] Jaberi SA, Cohen A, D’Souza C, Abdulrazzaq YM, Ojha S, Bastaki S, et al. Lipocalin-2: Structure, function, distribution and role in metabolic disorders. Biomed Pharmacother. 2021;142:112002. 34463264 10.1016/j.biopha.2021.112002

[5] Ceylan M, Erbak Yilmaz H, Narin F, Tatakis DN, Saglam M. Gingival crevicular fluid lipocalin-2 and semaphorin3A in stage III periodontitis: Non-surgical periodontal treatment effects. J Periodontal Res. 2022;57:724–32. 35468224 10.1111/jre.12995

[6] WHO. WHO’s Global oral health status report: towards universal health coverage for oral health by 2030. 2022. Available from: https://www.who.int/publications/i/item/9789240061484.

[7] Hajishengallis G, Chavakis T, Lambris JD. Current understanding of periodontal disease pathogenesis and targets for host-modulation therapy. Periodontology. 2020;84:14–34. 10.1111/prd.12331 PMC7457922 32844416

[8] Cardoso EM, Reis C, Manzanares-Céspedes MC. Chronic periodontitis, inflammatory cytokines, and interrelationship with other chronic diseases. Postgrad Med. 2018;130:98–104. 29065749 10.1080/00325481.2018.1396876

[9] Hajishengallis G. Immunomicrobial pathogenesis of periodontitis: keystones, pathobionts, and host response. Trends Immunol. 2014;35:3–11. 24269668 10.1016/j.it.2013.09.001 PMC3947349

[10] Gao S, Lin M, Chen W, Chen X, Tian Z, Jia T, et al. Identification of potential diagnostic biomarkers associated with periodontitis by comprehensive bioinformatics analysis. Sci Rep. 2024;14:93. 38168591 10.1038/s41598-023-50410-y PMC10761864

[11] Caton JG, Armitage G, Berglundh T, Chapple ILC, Jepsen S, Kornman KS, et al. A new classification scheme for periodontal and peri-implant diseases and conditions – Introduction and key changes from the 1999 classification. J Clin Periodontol. 2018;45:S1–S8. 29926489 10.1111/jcpe.12935

[12] Cafiero C, Spagnuolo G, Marenzi G, Martuscelli R, Colamaio M, Leuci S. Predictive Periodontitis: The Most Promising Salivary Biomarkers for Early Diagnosis of Periodontitis. J Clin Med. 2021;10:1488. 33916672 10.3390/jcm10071488 PMC8038382

[13] Bumm CV, Ern C, Folwaczny J, Wölfle UC, Heck K, Werner N, et al. Periodontal grading-estimation of responsiveness to therapy and progression of disease. Clin Oral Investig. 2024;28:289. 38691197 10.1007/s00784-024-05678-3 PMC11062956

[14] Ghallab NA. Diagnostic potential and future directions of biomarkers in gingival crevicular fluid and saliva of periodontal diseases: Review of the current evidence. Arch Oral Biol. 2018;87:115–24. 29288920 10.1016/j.archoralbio.2017.12.022

[15] Baltacioglu E, Sukuroglu E. Protein carbonyl levels in serum, saliva and gingival crevicular fluid in patients with chronic and aggressive periodontitis. Saudi Dent J. 2019;31:23–30. 30705565 10.1016/j.sdentj.2018.09.003 PMC6349948

[16] Lahdentausta LSJ, Paju S, Mäntylä P, Buhlin K, Tervahartiala T, Pietiäinen M, et al. Saliva and serum biomarkers in periodontitis and coronary artery disease. J Clin Periodontol. 2018;45:1045–55. 29972696 10.1111/jcpe.12976

[17] Nakajima M, Hosojima M, Tabeta K, Miyauchi S, Yamada-Hara M, Takahashi N, et al. β 2-Microglobulin and Neutrophil Gelatinase-Associated Lipocalin, Potential Novel Urine Biomarkers in Periodontitis: A Cross-Sectional Study in Japanese. Int J Dent. 2019;2019:10. 10.1155/2019/1394678 PMC6446109 31015837

[18] Heneberk O, Vernerova A, Kujovska Krcmova L, Wurfelova E, Radochova V. Neopterin Levels in Periodontitis and after Nonsurgical Periodontal Therapy: Evaluation of Gingival Crevicular Fluid, Oral Fluid, Serum and Urinary Samples—A Case-Control Study. Biomedicines. 2022;10:3200. 36551955 10.3390/biomedicines10123200 PMC9776342

[19] Ozmeriç N, Baydar T, Bodur A, Engin AB, Uraz A, Eren K, et al. Level of Neopterin, a Marker of Immune Cell Activation in Gingival Crevicular Fluid, Saliva, and urine in Patients With Aggressive Periodontitis. J Periodontol. 2002;73:720–5. 12146530 10.1902/jop.2002.73.7.720

[20] Pradeep AR, Nagpal K, Karvekar S, Patnaik K. Levels of lipocalin-2 in crevicular fluid and tear fluid in chronic periodontitis and obesity subjects. J Investig Clin Dent. 2016;7:376–82. 26097179 10.1111/jicd.12165

[21] Westerlund U, Ingman T, Lukinmaa P-L, Salo T, Kjeldsen L, Borregaard N, et al. Human neutrophil gelatinase and associated lipocalin in adult and localized juvenile periodontitis. J Dent Res. 1996;75:1553–63. 8906123 10.1177/00220345960750080601

[22] Aspiras MB, Barros SP, Moss KL, Barrow DA, Phillips ST, Mendoza L, et al. Clinical and subclinical effects of power brushing following experimental induction of biofilm overgrowth in subjects representing a spectrum of periodontal disease. J Clin Periodontol. 2013;40:1118–25. 24192073 10.1111/jcpe.12161

[23] Alfakry H, Malle E, Koyani CN, Pussinen PJ, Sorsa T. Neutrophil proteolytic activation cascades: a possible mechanistic link between chronic periodontitis and coronary heart disease. Innate Immun. 2016;22:85–99. 26608308 10.1177/1753425915617521

[24] Mahendra J, Mahendra L, divya D, Ilango P, Devarajan N, Thanigaimalai A. Association of Epstein–Barr virus, cytomegalovirus and lipocalin with periodontitis in type 2 diabetic subjects. Oral Dis. 2023;29:1163–71. 34850506 10.1111/odi.14091

[25] Isola G, Tartaglia GM, Santonocito S, Polizzi A, Williams RC, Iorio-Siciliano V. Impact of N-terminal pro-B-type natriuretic peptide and related inflammatory biomarkers on periodontal treatment outcomes in patients with periodontitis: An explorative human randomized-controlled clinical trial. J Periodontol. 2023;94:1414–24. 37433155 10.1002/JPER.23-0063

[26] Saaiq M, Ashraf B. Modifying “Pico” Question into “Picos” Model for More Robust and Reproducible Presentation of the Methodology Employed in A Scientific Study. World J Plast Surg. 2017;6:390–2. 29218294 PMC5714990

[27] von Elm E, Altman DG, Egger M, Pocock SJ, Gøtzsche PC, Vandenbroucke JP. Strengthening the Reporting of Observational Studies in Epidemiology (STROBE) statement: guidelines for reporting observational studies. BMJ. 2007;335:806–8. 17947786 10.1136/bmj.39335.541782.AD PMC2034723

[28] Wang L, Wang Y, Li Z, Yu B, Li Y. Unilateral versus bilateral pedicle screw fixation of minimally invasive transforaminal lumbar interbody fusion (MIS-TLIF): a meta-analysis of randomized controlled trials. BMC Surg. 2014;14:87. 25378083 10.1186/1471-2482-14-87 PMC4233064

[29] Jadad AR, Moore RA, Carroll D, Jenkinson C, Reynolds DJ, Gavaghan DJ, et al. Assessing the quality of reports of randomized clinical trials: is blinding necessary? Control Clin Trials. 1996;17:1–12. 8721797 10.1016/0197-2456(95)00134-4

[30] Alkayali MFMT, Badria FA, ElBaiomy AAB, Youssef JM. Effect of polycaprolactone nanofibers loaded with oxytetracycline hydrochloride and zinc oxide as an adjunct to SRP on GCF lipocalin-2 levels in periodontitis patients: A clinical and laboratory study. J Periodontol Implant Dent. 2022;14:76–83. 10.34172/japid.2022.024 PMC9871181 36714082

[31] Belstrøm D, Eiberg JM, Enevold C, Grande MA, Jensen CAJ, Skov L, et al. Salivary microbiota and inflammation-related proteins in patients with psoriasis. Oral Dis. 2020;26:677–87. 31916654 10.1111/odi.13277 PMC7188313

[32] Morelli T, Stella M, Barros SP, Marchesan JT, Moss KL, Kim SJ, et al. Salivary biomarkers in a biofilm overgrowth model. J Periodontol. 2014;85:1770–8. 25079398 10.1902/jop.2014.140180 PMC4383599

[33] Tan A, Gürbüz N, Özbalci F, Koşkan Ö, Yetkin Ay Z. Increase in serum and salivary neutrophil gelatinase-associated lipocalin levels with increased periodontal inflammation. J Appl Oral Sci. 2020;28:e20200276. 32997091 10.1590/1678-7757-2020-0276 PMC7521419

[34] Tsuchida S, Satoh M, Kawashima Y, Sogawa K, Kado S, Sawai S, et al. Application of quantitative proteomic analysis using tandem mass tags for discovery and identification of novel biomarkers in periodontal disease. Proteomics. 2013;13:2339–50. 23696425 10.1002/pmic.201200510

[35] Stathopoulou PG, Buduneli N, Kinane DF. Systemic Biomarkers for Periodontitis. Curr Oral Health Rep. 2015;2:218–26.

[36] Baser U, Oztekin G, Ademoglu E, Isik G, Yalcin F. Is the severity of periodontitis related to gingival crevicular fluid and serum high-sensitivity C-reactive protein concentrations? Clin Lab. 2014;60:1653–8. 25651710 10.7754/clin.lab.2014.131217

[37] Kamil TF, Ali OH. Association between The Cytokine IL-23 in Saliva with Periodontal Health and Disease. Egypt J Hospital Med. 2023;91:3920–4.

[38] Vahabi S, Yadegari Z, Pournaghi S. The comparison of the salivary concentration of interleukin-17 and interleukin-18 in patients with chronic periodontitis and healthy individuals. Dent Res J. 2020;17:280–6. PMC7688042 33282154

[39] Moura MF, Navarro TP, Silva TA, Cota LOM, Soares Dutra Oliveira AM, Costa FO. Periodontitis and Endothelial Dysfunction: Periodontal Clinical Parameters and Levels of Salivary Markers Interleukin-1β, Tumor Necrosis Factor-α, Matrix Metalloproteinase-2, Tissue Inhibitor of Metalloproteinases-2 Complex, and Nitric Oxide. J Periodontol. 2017;88:778–87. 28492359 10.1902/jop.2017.170023

[40] Rodvold JJ, Mahadevan NR, Zanetti M. Lipocalin 2 in cancer: When good immunity goes bad. Cancer Lett. 2012;316:132–8. 22075378 10.1016/j.canlet.2011.11.002

[41] Stallhofer J, Friedrich M, Konrad-Zerna A, Wetzke M, Lohse P, Glas J, et al. Lipocalin-2 Is a Disease Activity Marker in Inflammatory Bowel Disease Regulated by IL-17A, IL-22, and TNF-α and Modulated by IL23R Genotype Status. Inflamm Bowel Dis. 2015;21:2327–40. 26263469 10.1097/MIB.0000000000000515

[42] Elkhidir AE, Eltaher HB, Mohamed AO. Association of lipocalin-2 level, glycemic status and obesity in type 2 diabetes mellitus. BMC Res Notes. 2017;10:285. 28709459 10.1186/s13104-017-2604-y PMC5513122

[43] Ni J, Ma X, Zhou M, Pan X, Tang J, Hao Y, et al. Serum lipocalin-2 levels positively correlate with coronary artery disease and metabolic syndrome. Cardiovasc Diabetol. 2013;12:176. 24359145 10.1186/1475-2840-12-176 PMC3878105

[44] Viau A, El Karoui K, Laouari D, Burtin M, Nguyen C, Mori K, et al. Lipocalin 2 is essential for chronic kidney disease progression in mice and humans. J Clin Investig. 2010;120:4065–76. 20921623 10.1172/JCI42004 PMC2964970

[45] Martínez-García M, Hernández-Lemus E. Periodontal Inflammation and Systemic Diseases: An Overview. Front Physiol. 2021;12:709438. 10.3389/fphys.2021.709438 PMC8578868 34776994

