# Supplementary material for: A systematic review of the implications of lipocalin-2 expression in periodontal disease
Source: Evid Based Dent. 2024 Nov 8;26(1):69. doi: 10.1038/s41432-024-01070-y (PMC11953049; doi:10.1038/s41432-024-01070-y)
Supplement: Supplementary file 1 — SI Table 1 [file 41432_2024_1070_MOESM1_ESM.pdf]

SI Table 1. General characteristics of the included studies

Observational studies

Author, year and country	Purpose	Number of participants	Sex	Age (years)	Periodontal condition	BOP (%)	PD (mm)	CAL (mm)	PI	GI	Systemic disease	Sample evaluation method	Biological Fluid or tissue for detection	LCN2 levels	Related inflammatory biomarkers	Main outcome
Tsuchida et al., 2013 Japan	To identify new biochemical markers in GCF which predict the progression of periodontal diseases, through tandem mass tag labeling.	H:16<br>MIPD: 13<br>SPD: 18	21 F<br>26 M	43-46.3	Periodontal disease (mild, moderate, or severe) According to the classification of periodontal diseases by AAP, 1999.	NS	H: 1.1 ± 0.3<br>MIPD-MOPD : 3.7 ± 1.2<br>SPDe 7.6 ± 0.3	H: 1.2 ± 0.4<br>MIPD-MOPD : 3.8 ± 1.12<br>SPD: 7.7 ± 0.3	NS	H:0<br>MIPD-MOPD : 1 ± 0.5<br>SPD: 1.7 ± 0.4	No	Tandem mass tag labeling, western blot, and ELISA	GCF (ng/mL)	H: 5.6 ± 3.9<br>SPD:10.6 ± 2.7	Increased levels of MMP-9 in GCF.	TMT labeling identified MMP-9 and LCN2 and western blot analysis showed higher levels of both proteins in GCF of patients with severe periodontal disease than in healthy subjects.
Morelli et al., 2014 USA	To determine whether baseline salivary inflammatory biomarkers could change between different periodontal diseases stages over a 3-week stent induced biofilm overgrowth model.	BGI-H: 33<br>BGI-G: 34<br>BGI-P1: 33<br>BGI-P2: 34<br>BGI-P3:34	92 F<br>76 M	19-68	BGI- H: all PD <3 mm and BOP <10%.<br>BGI-), all PD <3 mm and BOP >10%.<br>3) BGI- P1, >1 site with PD >3 mm and BOP <10%.<br>4) BGI-P2, >1 site with PD >3 mm and BOP >10% but <50%.<br>5) BGI-P3, >1 site with PD >3 mm and BOP >50%.	BGI-H: 5.7 ± 1.3<br>BGI-G: 22.6 ± 1.4<br>BGI-P1: 6.9 ± 1.5<br>BGI-P2: 30 ± 1.4<br>BGI-P3: 59.5 ± 1.4	*Sites with PD > 4 mm*<br>BGI-H: 0<br>BGI-G: 0<br>BGI-P1: 2.2 ± 0.7<br>BGI-P2: 4.1 ± 0.6<br>BGI-P3: 7.8 ± 0.6	*Sites with PD > 3 mm*<br>BGI-H: 0.2 ± 0.7<br>BGI-G: 1.3 ± 0.8<br>BGI-P1: 1.8 ± 0.8<br>BGI-P2: 2.3 ± 0.8<br>BGI-P3: 5.1 ± 0.8	*Sites > 1 %*<br>BGI-H: 47.7 ± 3.7<br>BGI-G:63 ± 3.9<br>BGI-P1: 52.2 ± 4<br>BGI-P2: 76.7 ± 3.8<br>BGI-P3: 7.92 ± 3.9	*Sites > 1 %*<br>BGI-H: 65.7 ± 3.5<br>BGI-G: 74.7 ± 3.7<br>BGI-P1: 72.1 ± 3.8<br>BGI-P2: 94.3 ± 3.6<br>BGI-P3: 93.8 ± 3.7	No	Multiplex immunoassay	Saliva (log10)	BGI-H: 2.51 ± 0.05<br>BGI-G: 2.48 ± 0.05<br>BGI-P1: 2.60 ± 0.05<br>BGI-P2: 2.54 ± 0.05<br>BGI-P3: 2.75 ± 0.05	Increased IL-1β<br>MMP-3<br>MMP-8<br>MMP-9	Higher salivary levels of NGAL, IL-1β and diverse MMP-s in Stage III periodontitis compared with the other groups.
Pradeep et al., 2016 India	To determine LCN2 levels in GCF and tear fluid in	NOH: 10<br>OH: 10<br>NOCP: 10<br>OCP: 10	20 F<br>20 M	25-40	Chronic periodontitis (signs of gingival inflammation clinically (PD≥5mm,	NS	NOH: 1.9 ± 1.8<br>OH: 1.8 ± 0.78<br>NOCP:	NOH: 0<br>OH: 0<br>NOCP: 5.80 ± 1.54<br>OCP:	NS	NS	Obesity	ELISA	GCF (μg/L)<br>Tear fluid (ug/L)	*GCF*<br>NOH: 57.65 ± 6.4<br>OH: 80.9 ± 6.93	No	Increase in LCN2 levels in both gingival crevicular fluid and tear fluid in periodontitis groups. LCN2 further increased

	patients with obesity and chronic periodontitis.				GI>1, CAL≥3mm) and with bone loss seen radio-graphically		6.70 ± 1.60<br>OCP: 6.80 ± 1.540	6.0 ± 1.88						NOCP: 84.32 ± 6.46<br>OCP: 106.51 ± 9.382<br>*Tear Fluid*<br>NOH: 44.84 ± 6.4<br>OH: 72.6 ± 9.30<br>NOCP: 77.52 ± 8.73<br>OCP: 102.16 ± 9.08		when both diseases were present.
Nakajima et al.2019. Japan	To investigate the relationship between urinary biomarkers and periodontitis.	108	0 F<br>108 M	33-68	Moderate periodontitis	14.6 ±22.6	2.5 ±0.6	2.7 ±0.7	NS	NS	No	NS	Urine	NS	β-Macroglobulin is increased in patients with periodontitis	Urinary NGAL concentrations are positively correlated with periodontal parameters.
Tan et al., 2020. Turkey	To determine serum and salivary levels of NGAL and its correlation with IL-10 and IL-1 β.	H: 20<br>G: 20<br>GS1: 20<br>GS3: 20	35 F<br>45 M	25-63	Gingivitis, Stage I generalized periodontitis, Stage III generalized periodontitis.	H: 6 ±3<br>G:97 ±5<br>GS1: 99 ±2<br>GS3: 100 ±0	H: 1.34 ±0.17<br>G: 2.09 ±0.20<br>GS1:2.72 ±0.26<br>GS3: 3.80 ±0.89	H: 0<br>G: 0<br>GS1: 3.09 ±0.41<br>GS2: 4.61 ±1.35	H: 0.39 ±0.16<br>G: 1.80 ±0.36<br>GS1:1.93 ±0.15<br>GS3: 2.15 ±0.20	H: 0.33 ±0.13<br>G: 1.77 ±0.25<br>GS1: 1.90 ±0.11<br>GS3:2.15 ±0.20	No	ELISA	Serum and salivary (pg/L)	*Saliva*:<br>H: 158.32<br>G:194.9<br>GS1:208.47<br>GS3: 242.27<br><br>*Serum:*<br>H: 340.58<br>G:383.71<br>GS1:411.31<br>GS3: 510.44	IL-1β and IL-10 concentrations were higher in gingivitis than in generalized periodontitis.	Serum and salivary NGAL levels are proportionally increased with disease severity.
Belstrøm et. al., 2020 Denmark	characterize the composition of the salivary microbiota and quantify	PS: 27<br>P: 58<br>H: 52	61 F<br>76 M	38-80	Stage III periodontitis BOP ≥ 25% of total sites, with minimum two teeth with clinical	PS: 63<br>P: NS<br>H: NS	PS: 3.0<br>P: NS<br>H: NS	PS:2.8<br>P: NS<br>H: NS	PS: 82<br>P: NS<br>H: NS	NS	PS	Immunoassay	Saliva (ng/mL)	PS: 996 ± 320<br>P: 2072 ± 295<br>H:2551 ± 345	Transferrin is lower in psoriasis	Lower LCN2 concentrations in saliva of patients with psoriasis than in periodontitis.

	salivary levels of NGAL and transferrin in patients with psoriasis and periodontitis.				attachment level ≥ 4 mm and a minimum two teeth with PPD ≥ 6 mm											
Mahendra et al., 2021. India	To evaluate and compare lipocalin, adiponectin and periodontal viruses in the generalized periodontitis patients with and without DM.	GP: 35<br>GP+DM: 35	NS	35-60	Generalized periodontitis	NS	GP: 5.87 ±0.64<br>GP+DM: 6.02 ±0.58	GP: 6.51 ±0.82<br>GP+DM: 7.20 ±0.67	GP: 1.50 ±0.47<br>GP+DM 2.07 ±0.24	NS	Type 2 diabetes	ELISA	Subgingival tissue	GP: 700.99 ± 88.76<br>GP+DM: 1007.74 ±114.13	Adiponectin is even lower in patients with both GP +DM	An increased prevalence of periodontal viruses, and LCN2 levels was observed in patients with diabetes and periodontitis compared with patients with only diabetes.

Experimental studies

**Author, year and country**	**Type of study and purpose**	**Number of participants**	**Sex**	**Age (years)**	**Periodontal condition**	**Treatment**	**Biological Fluid for LCN2 detection**	**BOP (%)**	**PD (mm)**	**CAL (mm)**	**PI**	**Sample evaluation method**	**GI**	**LCN2 (baseline)**	**LCN2 (after treatment)**	**Related inflammatory biomarkers**	**Main outcome**
Aspiras et al., 2013 USA	Investigate the short-term effects of power brushing following experimental induction of biofilm overgrowth in periodontal disease states.	H: 33<br>G: 34<br>P1: 33<br>P2: 34<br>P3: 34	112 F<br>56 M	18-75	Gingivitis, Periodontitis stage I, II and III	Manual brushing and Sonicare	Saliva [log10]	Day 0: 31.7 ±19.6<br>Day 35: 29.0 ±16.5<br>*Data for all subjects	Day 0: 2.19 ±0.39<br>Day 35: 2.16 ±0.38<br>*Data for all subjects	Day 0: 1.13 ±0.30<br>Day 35: 1.11<br>*Data for all subjects	Day 0: 0.79 ±0.41<br>Day 35: 0.78 ±0.37<br>*Data for all subjects	Bead-based multiplexing analysis	Day 0: 0.89 ±0.31<br>Day 35: 0.87 ±0.30<br>*Data for all subjects	NS	Manual brushing:2.58[log10]<br>Sonicare: 2.61[log10] 0.38	IL-1β	Power brushing with Sonicare reduced plaque, bleeding, and improved periodontal status by decreasing IL-1β

																	after 2 weeks but it did not induce significant changes in NGAL salivary concentrations .
Isola et al., 2023<br>Italy	To assess the efficacy of periodontal treatment in NT-proBNP and CVD related biomarkers.	Control: 24<br>Test: 24	24 F<br>24 M	35-70	Stage III periodontitis	Full-mouth scaling and root planning	Serum	*Baseline:*<br>Control :48.4 ±15.1<br>Test: 47.2 ±16.5<br><br>*6 months:*<br>Control: 24.3 ± 17.1<br>Test: 18.1 ± 15.6	*Baseline:*<br>Control: 4.95 ±0.3<br>Test: 4.88 ±0.4<br>*6 months:*<br>Control: 3.59 ±0.5<br>Test: 2.71 ±0.4	*Baseline:*<br>Control: 5.21 ±0.3<br>Test 5.15 ±0.3<br><br>*6 months:*<br>Control: 3.87 ±0.5<br>Test: 2.75 ±0.3	*Baseline:*<br>Control: 38.6 ±12.2<br>Test 39.1 ±15.4<br><br>*6 months:*<br>Control: 21.2 ± 15.6<br>Test: 17.3 ±14.2	ELISA	NS	Control: 479.6 ±16.2<br>Test: 477.6 ±13.5	Control: 468 ± 12.5<br>Test: 452.6 ± 11.4	Hs-CRP<br>NT-proBNP.	After 6 months, FM-SRP was more effective than SOC in reducing periodontal parameter and NGAL concentrations. nd other biomarkers like hs-CRP and NT-proBNP.
Ceylan et al., 2022<br>Turkey	To evaluate GCF LCN2, Sema3A and TNF-α in patients with gingivitis and periodontitis and their changes	H: 20<br>G: 20<br>P: 20	27 F<br>33 M	18-70	Stage III periodontitis	Non-surgical periodontal treatment	GCF (ng/mL)	*Baseline:*<br>H: 7.2 ± 3<br>G: 58.3 ± 24.8<br>P: 69.5 ± 19.7<br><br>*3 months*<br><br>P: 37.1 ± 13.2	*Baseline:*<br>H: 1.6 ± 0.1<br>G: 2.0 ± 0.3<br>P: 3.3 ± 0.5<br>*3 months*<br><br>P: 2.3 ± 0.4	*Baseline:*<br>H: 0<br>G: 0<br>P: 3.7 ± 1<br><br>*3 months:*<br>P: 3 ± 1	*Baseline:*<br>H: 0.6 ± 0.2<br>G: 1.6 ± 0.6<br>P: 1.6 ± 0.5<br>*3 months:*<br>P: 1 ± 0.3	ELISA	*Baseline:*<br>H: 1.0 ± 1<br>G: 1.6 ± 0.4<br>P: 1.8 ± 0.3<br>*3 months:*<br>P: 1.4 ± 0.1	H: 1.7 ± 1<br>G: 6.2 ± 2.9<br>P: 4.9 ± 1.9	H: NS<br>G: NS<br>P: 2.7 ± 0.7	TNF-α<br>Sema3A	LCN2 and TNF-α levels are higher in periodontitis and decreased after treatment.

	after NSPT.																
Alkayali et al., 2022 Egypt	To assess the use of PCL nanofibers loaded with OTRC and ZnO as an adjunct mechanical therapy for periodontitis.	PCL+OTC + XNO: 10<br>PCL+OTC: 10<br>PCL+ZNO: 10<br>PCL: 10<br>SRP: 10<br>H: 10	30 F<br>30 M	30-60	Stage II grade A	Scaling and root planing vs. polycarprolactone nanofibers with oxytetracycline or zinc oxide.	GCF	NS	*Before:*<br>PCL+OTC+ZNO: 3.30 ± 0.27<br>PCL+OTC: 3.25 ± 0.34<br>PCL+ZNO: 3.27 ± 0.37<br>PCL: 3.39 ± 0.348<br>SRP: 3.11 ± 0.38<br>H: 1.4 ± 0.017<br>*After:*<br>PCL+OTC+ZNO: 1.565± 0.17<br>PCL+OTC: 1.60 ± 0.175<br>PCL+ZNO: 1.67± 0.244<br>PCL: 2.82± 0.352<br>SRP: 2.19 ± 0.35<br>H: 1.464 ± 0.02	*Before:*<br>PCL+OTC+ZNO: 2.80± 0.236<br>PCL+OTC: 2.57 ± 0.33<br>PCL+ZNO: 2.78 ± 0.31<br>PCL: 2.72 ± 0.302<br>SRP: 2.62 ± 0.289<br>H: 0<br>*After:*<br>PCL+OTC+ZNO: 1.31± 0.137<br>PCL+OTC: 1.47± 0.24<br>PCL+ZNO: 1.53± 0.16<br>PCL: 2.149± 0.33<br>SRP: 2.03 ± 0.36<br>H: 0.0± 0.0	*Before:*<br>PCL+OTC+ZNO: 2.34± 0.28<br>PCL+OTC: 2.49 ± 0.19<br>PCL+ZNO: 2.33±0.21<br>PCL: 2.41 ± 0.30<br>SRP: 2.45 ± 0.09<br>H: 0.16 ± 0.06<br>*After:*<br>PCL+OTC+ZNO: 0.809± 0.085<br>PCL+OTC: 0.850± 0.08<br>PCL+ZNO: 0.821± 0.081<br>PCL: 0.872± 0.064<br>SRP: 0.865 ± 0.055<br>H: 0.167 ± 0.07	ELISA	*Before:*<br>PCL+OTC +ZNO: 1.84± 0.17<br>PCL+OTC: 1.82 ± 0.18<br>PCL+ZNO: 1.79 ± 0.22<br>PCL: 1.86 ± 0.309<br>SRP: 1.75 ± 0.159<br>H: 0.19 ± 0.01<br>*After:*<br>PCL+OTC +ZNO: 0.331± 0.049<br>PCL+OTC: 0.341 ± 0.0.058<br>PCL+ZNO: 0.353± 0.072<br>PCL: 0.744± 0.195<br>SRP: 0.434± 0.129<br>H: 0.191 ± 0.06	PCL+OTC+ZNO: 247.5± 22.1<br>PCL+OTC: 252.4 ± 20.36<br>PCL+ZNO: 226 ± 47.39<br>PCL: 240.10 ± 37.4<br>SRP: 233.6 ± 31.2<br>H: 187.3 ± 14.8	PCL+OTC +ZNO: 180.9± 23.87<br>PCL+OTC: 183.20 ± 6.08<br>PCL+ZNO: 185.6 ± 12.42<br>PCL: 219.3 ± 40.3<br>SRP: 220.7 ± 12.67<br>H: 187.3 ± 14.8	No	LCN2 levels are increased in periodontitis at baseline. Clinical parameters of periodontitis and GCF LCN2 levels improved after all treatments, being PCL+OTC+ZNO the most effective option.

BOP: Bleeding on probing, PD: probing depth, CAL: clinical attachment loss, PI: plaque index, GI: gingival index, H: periodontally healthy, G: gingivitis, P: periodontitis, F: Female, M: Male, GCF: gingival crevicular fluid, NS: Not specified, USA: United States of America, NGAL: Neutrophil gelatinase associated lipocalin, BGI-H: Biofilm gingival interface, GI-Healthy, BGI-G: Biofilm gingival interface-gingivitis, BGI-P1: Biofilm gingival interface-periodontitis stage I, BGI- P2: Biofilm gingival interface-periodontitis stage II, BGI-P3: Biofilm gingival interface-periodontitis stage III, MIPD: Mild periodontal disease, MOPD: Moderate periodontal disease, SPD: Severe periodontal disease, AAP: American Academy of Periodontology, NOH: Non-obese healthy, OH: Obese healthy, NOCP: Non-obese chronic periodontitis, OCP: Obese chronic periodontitis, Psoriasis: PS, DM: diabetes mellitus, NT-proBNP: N-terminal portion of the B-type natriuretic propeptide, CVD: cardiovascular disease, PCL+OTC+ZNO: Polycarprolactone nanofibers and oxytetracycline and zinc oxide, PCL+OTC: Polycarprolactone nanofibers and oxytetracycline, PCL+ZNO: Polycarprolactone nanofibers and zinc oxide, PCL: Polycarprolactone nanofibers.
